# Supplementary material for: A cross-kingdom conserved ER-phagy receptor maintains endoplasmic reticulum homeostasis during stress
Source: eLife. 2020 Aug 27;9:e58396. doi: 10.7554/eLife.58396 (PMC7515635; doi:10.7554/eLife.58396)
Supplement: Supplementary file 6. [file elife-58396-supp6.docx]

**Table S6.** Summary of thermodynamic parameters of the interactions studied in this paper:

| **Name** | **N (sites)** | ***K*_D_ (nM)** | **∆H (kcal/mol)** | **∆G (kcal/mol)** | **-T∆S (kcal/mol)** |  |
| --- | --- | --- | --- | --- | --- | --- |
| **ATG8A** | | | | | | |
| *At*C53 IDR | 0.588 ± 0.008 | 907 ± 124 | -4.11 ± 0.07 | -8.25 | -4.14 |  |
| *AIMwt* | 0.858 ± 0.040 | 700± 421 | -1.19 ± 0.08 | -8.40 | -7.21 |  |
| *AtAIM* | 1.090 ± 0.068 | 9880± 2490 | -1.97 ± 0.17 | -6.83 | -4.87 |  |
| **GABARAP** | | | | | | |
| *Hs*C53 IDR | 0.541 ± 0.028 | 8040 ± 1410 | -9.53 ± 0.75 | -6.95 | 2.57 |  |
| *AIMwt* | 1.070 ± 0.009 | 157± 41 | -4.30 ± 0.07 | -9.29 | -4.99 |  |
| *HsAIM* | 1.220 ± 0.035 | 9910± 1300 | -5.05 ± 0.21 | -6.83 | -1.78 |  |
